# Cerebral Venous Sinus Thrombosis in COVID-19: An Unusual Presentation

**DOI:** 10.7759/cureus.13767

**Published:** 2021-03-08

**Authors:** Ikwinder Kaur, Charmee Vyas, Mohsin Mughal, Haresh Gandhi, Doantrang Du

**Affiliations:** 1 Internal Medicine, Monmouth Medical Center, Long Branch, USA; 2 Medicine, Monmouth Medical Center, Long Branch, USA; 3 Internal Medicine, Robert Wood Johnson (RWJ) Barnabas Health, Long Branch, USA

**Keywords:** corona virus disease 2019, thrombo embolic disease, cerebral venous sinus thrombosis (cvst)

## Abstract

Severe acute respiratory syndrome-coronavirus 2 (SARS-CoV-2) has been associated with a significantly increased risk of venous and arterial thromboembolism, particularly in severely sick patients. Recently, cerebral venous sinus thrombosis (CVST) cases have been reported in the context of coronavirus disease-2019 (COVID-19). These cases either had an active COVID infection with a positive reverse transcription‐polymerase chain reaction (RT‐PCR) or were symptomatic (fever, respiratory symptoms, myalgia) during the presentation. We present here a 41-year-old male with CVST who had negative RT-PCR and positive immunoglobulin G (IgG) COVID-19 antibodies. He was neither diagnosed nor had a flu-like illness before admission. This case highlights that CVST can be a late sequela of previously undiagnosed asymptomatic COVID-19 infection.

## Introduction

Coronavirus disease-2019 (COVID-19) has been associated with a heightened risk of arterial and venous thromboembolic complications including stroke, myocardial infarction, acute limb ischemia, and venous thromboembolism. The proposed mechanisms include direct viral endothelial invasion followed by activation of the coagulation cascade, cytokine storm-related prothrombotic state, and/or increased platelet activity. Various virus infections have been associated with coagulation disorders including herpes simplex, human immunodeficiency virus (HIV), cytomegalovirus (CMV), severe acute respiratory syndrome (SARS), and Middle East respiratory syndrome (MERS) [1]. Cerebral venous sinus thrombosis (CVST) is a rare cause of stroke, reported recently in context with severe acute respiratory syndrome-coronavirus 2 (SARS-CoV-2). The clinical presentation of CVST varies from acute to subacute onset of headache, altered mental status, seizures, and/or focal deficits. Magnetic resonance imaging (MRI) or magnetic resonance venography (MRV) is the preferred imaging modality due to its high sensitivity. Computed tomography venography (CTV) is a reliable option when MRI is contraindicated. The gold standard cerebral angiography is performed when other modalities are inconclusive or unavailable [2]. Early identification and prompt treatment with anticoagulation and/or endovascular therapies are critical to prevent progression to cerebral edema, thus improving outcomes.

## Case presentation

**Figure 1:**
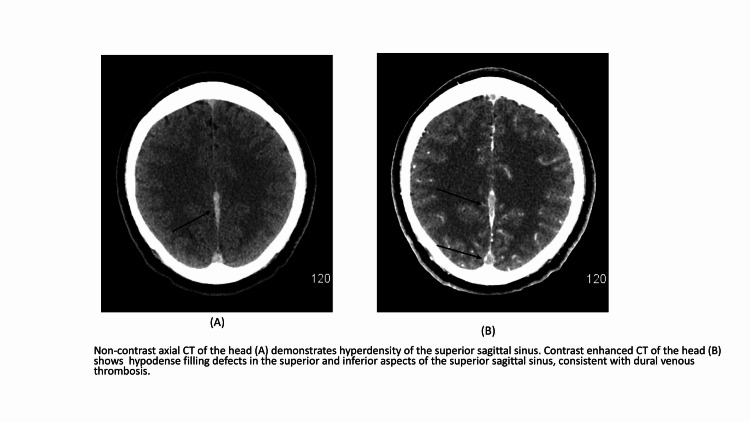
Cerebral venous sinus thrombosis (CVST) radiological image

A 41-year-old right-handed male with no significant past medical history presented with sudden onset of severe frontal headache started on the day of admission. He denied any aggravating or relieving factors. It was associated with progressively worsening difficulty in speaking started 1-2 hours after the beginning of the headache. He denied vision loss, focal weakness, sensory loss, vomiting, abnormal body movements, involuntary loss of urine or stool, and loss of consciousness. The patient was afebrile at admission and remained afebrile throughout hospitalization. Other vital signs at admission were - blood pressure 141/76, heart rate 77, respiratory rate 18, and oxygen saturation 97% on room air. Physical examination was remarkable for significant expressive aphasia without other neurological deficits. Laboratory data showed elevated erythrocyte sedimentation rate (ESR) (29mm/hr; normal 0-15 mm/hr) and c-reactive protein (CRP) (44.89 mg/L; normal<7) on admission. SARS-CoV-2 reverse transcription‐polymerase chain reaction (RT‐PCR) was negative, immunoglobulin G (IgG) antibodies were positive. Complete blood count (CBC), comprehensive metabolic panel (CMP), and lipid panel were within normal limits. Imaging (CT scan, MRI scan, and MRV) of the head was consistent with an acute dural sinus thrombosis of the superior sagittal sinus extending into the left transverse and sigmoid sinus (Figures 1A-1B). Also, contrast filling defects in the straight sinus, the vein of Galen, and in the right cortical vein at the vertex were noted. There was no evidence of intracranial haemorrhage, infarction, mass effect, or cerebral edema. The patient was immediately started on the intravenous heparin infusion. The patient’s symptoms (aphasia) improved significantly over 3-4 hours and were completely resolved in the next two days. During the hospital stay, EEG showed potential left temporal seizure focus, and the patient was started on prophylactic antiepileptics. The hypercoagulable workup was unremarkable and is summarized in Table 1. Other investigations including HIV screen and antinuclear antibody (ANA) screen were negative. Due to the absence of established risk factors, COVID-19 was considered as a potential etiology in our patient. The patient was discharged on enoxaparin on day 4 for further follow up with a multidisciplinary team as an out-patient.

**Table 1:** Hypercoagulable work-up

Hypercoagulable work-up	Value	Reference range
Protein S activity (%)	93	70-150
Protein C activity (%)	85	70–180
Antithrombin III assay (%)	106	80–135
Factor V Leiden mutation	Not detected	
Prothrombin gene (G20210A) mutation	Not detected	
Lupus anticoagulant	Not detected	
Anticardiolipin IgG (GPL)	Negative (<14)	
Factor VIII assay (%)	161.8	50-200

## Discussion

CVST is a rare neurovascular emergency caused by several systemic or local risk factors. These include hematologic prothrombotic states, infections, malignancies, pregnancy, hormonal contraceptives, dehydration, head injury, and connective tissue diseases. Prothrombotic state induced by hyperactivation of cytokines in COVID-19 has been associated with arterial and venous thrombosis, especially in severely sick patients [3,4]. The true prevalence of CVST with COVID-19 has not been discussed to date. In a review article, the majority of patients were middle-aged and had a mild-moderate disease [3]. Clinical manifestations are highly variable and include acute to subacute onset of severe headache, seizures, focal deficits, or encephalopathy. So far, all reported cases had an active COVID-19 infection. To our knowledge, one case report had a patient with negative RT-PCR and positive IgG antibodies. However, the patient had fever and respiratory symptoms before presenting with neurological symptoms [5]. Interestingly, our patient was immune (positive SARS-CoV-2 IgG antibodies) at presentation and had no respiratory symptoms before admission. The mainstay of the treatment is therapeutic anticoagulation. The survival rate reaches 60% with early initiation of anticoagulation [3]. In this case, the patient was not considered a candidate for thrombectomy due to significant improvement of neurological symptoms.

## Conclusions

Our case reveals that a hypercoagulable state can be seen as a late manifestation even if the patient is immune after an asymptomatic COVID-19 infection. All patients with CVST should be tested for COVID-19 during the current pandemic, regardless of symptoms. Due to potential association, COVID-19 should be considered as one of the differentials for CVST.
